# Prevalence of Self Medication Practice among Dental Undergraduates in a Dental college

**DOI:** 10.31729/jnma.4740

**Published:** 2020-01-31

**Authors:** Aastha Shrestha, Nirjala Laxmi Madhikarmi

**Affiliations:** 1 Department of Pharmacology, Kantipur Dental College, Basundhara, Kathmandu, Nepal; 2 Department of Biochemistry, Kantipur Dental College, Basundhara, Kathmandu, Nepal

**Keywords:** *dental students*, *prevalence*, *self medication*, *undergraduate*

## Abstract

**Introduction:**

Self-medication practice among future prescribers can cause a serious threat to the health care profession. There has been an increasing trend among medical and dental students for self-medication. The objective of our study was to find the prevalence and practice of self-medication among dental undergraduates in Kantipur Dental College and Teaching Hospital.

**Methods:**

A descriptive cross-sectional study was conducted among all the dental undergraduate students of Kantipur Dental College, Kathmandu, from July to September 2018. Ethical clearance was obtained from the institutional review board. A convenience sampling method was used. A prevalidated questionnaire was handed to the students in their classroom to collect the data. The data were analyzed using Statistical Package for the Social Sciences version 16 and Microsoft Excel 2010 and presented as frequency and percentage.

**Results:**

The prevalence of self-medication among dental undergraduates was found to be in 150 (83.3%) out of a total of 180 students who participated in the study.

**Conclusions:**

Self-medication was commonly practiced by dental students. Self-medication should be considered as a serious threat, especially among the students with inadequate knowledge of drug, dose, and duration of treatment.

## INTRODUCTION

Self-medication is the use of drugs by the individuals to treat the self-diagnosed disorders or symptoms, where the medicines are used intermittently or continuously on their own or with the help of pharmacists or media, but without proper advice from medical professionals.^1^ Selfmedication practice is increasing worldwide, especially in developing countries.^1,2^ Nepal being a developing country where access to medical service is difficult and shortage of medical personnel especially in rural areas, self-medication becomes a better alternative for treating common ailments.^3^

The students, especially medical and dental undergraduates are more involved in the self-medication practice as they are empowered with good knowledge of drugs, diseases and have greater access to medicine.^4^ Recent studies have shown a greater prevalence of selfmedication among medical and dental students.^5,6^

The objective of our study was to determine the prevalence of self-medication practice among dental students.

## METHODS

A descriptive cross-sectional study was conducted among the dental undergraduates of Kantipur Dental College and Teaching Hospital (KDCTH). The study was conducted between July and September 2018. Ethical clearance was obtained from the institutional review board of KDCTH. The students who practiced selfmedication during the last six months were included in the study. Students who were absent during the data collection and those who were not willing to participate were excluded from the study. The convenience sampling method was used to collect the data and the sample size was calculated using the formula.

$$\begin{array}{ll} \text{n} & \text{= }{\text{Z}}^{\text{2}}\text{p}(\text{1}-\text{ p)}/{\text{e}}^{2}\\ & \text{= }{(\text{1.96)}}^{\text{2}}\times \text{0.88}\times \text{(1}-\text{0.88)}/{(\text{0.05)}}^{\text{2}}\\ & \text{= }162.2 \end{array}$$

where,

n = minimum required sample size
Z = 1.96 at 95% Confidence interval
p = prevalence from a previous study (88%)^6^
1-p = 12%
e = margin of error as 5%

The total sample size calculated from the above formula was obtained as 162.2. Taking a non-response rate of 10%, the calculated sample size was 180.

The Bachelor of Dental Surgery students from the first year to the final year were approached after their first morning lecture. They were explained about the nature and purpose of the study. The questionnaire was distributed, and appropriate instructions were given for filling up. The questionnaire consisted of both open- ended and closed-ended questions, which contain three parts; section A consists of demographic details of the students such as age, gender, year of BDS, mother's, and father's profession, whether they self-medicated in the last six months. If their answer was affirmative to self-medication, they were instructed to fill section B, which included a question regarding the practice of self-medication. Section C comprised of question concerning their attitude toward self-medication. The student who did not self-medicate were instructed to fill only section C. The collected data were entered, and descriptive analyses were done using SPSS version 16 and MS-EXCEL 2010.

## RESULTS

Out of 180 students, 150 (83.3%) of them practiced self-medication. Among the total students, i.e., 39 (21.7%) were male and 141 (78.3%) were female students, the tendency of self-medication practice was found to be 29 (19.3%) in male and 121 (80.6%) in female students (Table 1).

**Table 1:** Gender distribution in self-medication practice.

Gender	n (%)
Male	29 (19.3)
Female	121 (80.7)
Total	150 (100)

Among the total participants from all the BDS year students 41 (22.7%), 46 (25.5%), 39 (21.7%), 36 (20%) and 18 (10%) students from BDS I, BDS II, BDS III, BDS IV and BDS V year respectively, self-medication practice was seen common among the students of BDS II 40 (26.7%) and BDS I 35 (23.3%) (Table 2).

**Table 2:** Year-wise distribution of prevalence of self-medication practice.

Year of study	n (%)
BDS I year	35 (23.3)
BDS II year	40 (26.7)
BDS III year	34 (22.7)
BDS IV year	25 (16.7)
BDS final year	16 (10.7)
Total	150 (100)

The major indication for practicing self-medication was common cold and cough 75 (50%). Among the students who practiced self-medication, analgesic was commonly used drugs 77 (51.4%) and the source of information for the drug selection was their own pharmacology knowledge 77 (51.4%). It was found that drowsiness was the common side effect experienced by the participants 22 (14.7%).

## DISCUSSION

The prevalence of self-medication among dental students was 83.3%, which is consistent with the study conducted in western Nepal, 81.9%, and 84% in Karnataka, India.^4,8^ Our finding for the prevalence was higher compared to other studies: 48.3% in Eastern Nepal, 40.9% in Pune, 57.05% in West Bengal, 65% in Kolkata and 71.7% in Nagpur, India.^8–12^ Variation has been seen on the prevalence of self-medication which may be due to the factor like a difference in the study population (medical and dental students), availability of the drug in local pharmacy without valid prescription, availability of over the counter (OTC) drugs, and acquired knowledge of medicine, convenience and time-saving.

Self-medication practice is generally expected to increase from the first year to final year students. As they reach the final year, a student develops confidence in the knowledge about drugs, disease, and about prescribing the drug. Studies have evidenced that the prevalence of self-medication increases with the increase in the year of education. Banerjee et al. reported proportionate increase in the practice of self-medication 41.7% first year to 79.31% final year.^10^ Correspondingly the proportion was 40.5% in the first year and 90.80% in the fourth year in a study conducted by Pal et al. and 86.33% in the first year and 91.75% in final year in a study conducted by Patil et al.^6,11^ In this study selfmedication practice was higher among the second year compared to final year students. The low prevalence in our study could be the awareness of the potential disadvantage of self-medication like side effects, drug interactions, or maybe due to under-reporting in the final year students. The high prevalence in second and first-year students could be the pharmacology subject which is taught as a basic course in first and second year BDS because of which they gain knowledge about drugs and are highly enthusiastic about using them in day to day life.

In our study, common cold and cough were the most common indication for practicing self-medication, and similar observations were seen in other studies.^6,8,9,13^ However, fever was a common indication for selfmedication in other studies.^11,12,14^ The common reason for self-medication was illness being too minor to visit the doctor. This might be attributed to the ignorance and lack of knowledge on the progression of the disease. As many diseases may initially appear to be mild, but misdiagnosis and wrong treatment may cause many serious issues. The finding was consistent with the studies conducted by Kasulkar A et al, Lukovic J et al, Kalra DD et al, and Sarraf DP et al.^8,9,12,15^ In contrast to our study, convenience, time-saving and other's advice were the common reason for self-medication in other studies.^7,13,16^

Analgesics were the commonly used drug as selfmedication in our study, which is consistent with the studies done by Nirmal et al. and Mehta et al.^7,17^ Studies conducted in Pune, Mangalore, and Ethiopia antipyretics were frequently used for self-medication.^9,14,18^ However, antibiotic was common self-medication in a study conducted in Karnataka and Serbia,^6,15^ but the use of antibiotics was much lower 6.7% in our study. This may be due to the current trends in the awareness program of antibiotic resistance.

Most of the students selected the drug for selfmedication with their knowledge from pharmacology. The study was incongruent to study conducted by Kumar et al. in Mangalore.^14^ In contrast to our study, media, and magazine, seniors, use of old prescription for the same illness and textbooks were the guiding source of information for drug selection.^6,8,9^

The majority of the students, 65% accepted selfmedication as a part of self-care. A similar finding was reported in other studies,^8,13^ whereas the acceptance rate was higher than that reported by Kumar et al.^14^ Though the self-medication is a part of self-care, it is considered safe only when medicines are used judiciously. Irrational use of drugs, even for OTC medicines, can cause a hazardous effect on human health. The majority of the students agreed that self-medication is harmful if taken without proper knowledge of drugs and disease. The practice of self-medication should be initiated only when an individual has appropriate knowledge about medicine. Inappropriate self-medication can have a number of potential risks, for example, drug interactions with the prescribed medicines, drug contraindications, inappropriate duration of use of medicine, and risk of drug dependence and abuse.

The present study has certain limitations. The data were collected only among dental students from a private dental college due to which result obtained cannot be generalized. All the students were encouraged to fill up the questionnaire independently, but mutual influence cannot be ruled out. Moreover, the study was based on the self-medication practice in the preceding six months; there is a higher chance of recall bias among the students. Multicentric studies should be carried out among the student of different field, including the general population to understand the various factors influencing the practice of self-medication. Along with that, strong policies should be applied to prohibit the supply of medicines without a valid prescription.

## CONCLUSIONS

The self-medication practice was high among the dental undergraduates. Cold and cough was the most common indication for practicing self-medication. Analgesics and antipyretics were the most commonly used drug for self-medication. The mild nature of the illness was the reason for practicing self-medication. Educational intervention about the responsible self-medication and potential health risk should be implemented to decrease the rate of the irrational practice of self-medication.
